# Endobronchial Enigma: A Clinically Rare Presentation of *Nocardia beijingensis* in an Immunocompetent Patient

**DOI:** 10.1155/2015/970548

**Published:** 2015-12-24

**Authors:** Nader Abdel-Rahman, Shimon Izhakain, Walter G. Wasser, Oren Fruchter, Mordechai R. Kramer

**Affiliations:** ^1^The Pulmonary Institute, Rabin Medical Center, Beilinson Hospital, 49100 Petah Tikva, Israel; ^2^The Sackler Faculty of Medicine, Tel Aviv University, 69978 Tel Aviv, Israel; ^3^Mayanei HaYeshua Medical Center, 51544 Bnei Brak, Israel; ^4^Rambam Health Care Campus, 3109601 Haifa, Israel

## Abstract

Nocardiosis is an opportunistic infection caused by the Gram-positive weakly acid-fast, filamentous aerobic Actinomycetes. The lungs are the primary site of infection mainly affecting immunocompromised patients. In rare circumstances even immunocompetent hosts may also develop infection. Diagnosis of pulmonary nocardiosis is usually delayed due to nonspecific clinical and radiological presentations which mimic fungal, tuberculous, or neoplastic processes. The present report describes a rare bronchoscopic presentation of an endobronchial nocardial mass in a 55-year-old immunocompetent woman without underlying lung disease. The patient exhibited signs and symptoms of unresolving community-acquired pneumonia with a computed tomography (CT) scan that showed a space-occupying lesion and enlarged paratracheal lymph node. This patient represents the unusual presentation of pulmonary* Nocardia beijingensis* as an endobronchial mass. Pathology obtained during bronchoscopy demonstrated polymerase chain reaction (PCR) confirmation of nocardiosis. Symptoms and clinical findings improved with antibiotic treatment. This patient emphasizes the challenge in making the diagnosis of pulmonary nocardiosis, especially in a low risk host. A literature review presents the difficulties and pitfalls in the clinical assessment of such an individual.

## 1. Introduction

*Nocardia* infection was initially reported by Nocard, a French veterinarian in 1888 [1], who described an uncommon Gram-positive bacterial infection caused by aerobic Actinomycetes. Currently there are 85 identified species of* Nocardia* classified by using 16S rRNA gene sequence; approximately 25 species are associated with human infections. These include* Nocardia asteroides* complex (more than 50% human cases),* N. brasiliensis*,* N. abscessus*,* N*.* cyriacigeorgica*,* N. farcinica*,* N. nova*,* N. transvalensis* complex,* N. nova* complex,* N. pseudobrasiliensis*,* Nocardia veteran*,* N. cerradoensis* [2], and recently reported* N. beijingensis* [3–8]. Sputum isolation of* Nocardia* always represents an infection since* Nocardia* is not part of the human normal flora.

The clinical presentation of pulmonary nocardiosis can be acute, subacute, or chronic pneumonia. The diagnosis can be challenging, as often signs and symptoms are nonspecific including fever, night sweats, fatigue, anorexia, weight loss, dyspnea, cough, hemoptysis, and pleuritic chest pain [9, 10]. Moreover, there are a wide range of radiographic presentations such as lobar infiltrates, effusion, abscesses, cavities, lobar consolidations, subpleural plaques, and masses.

Nocardiosis has been observed to be associated with a wide range of conditions, especially those with impaired cell mediated immunity, including solid organ and hematopoietic stem cell transplantation, acquired immunodeficiency syndrome (AIDS), and hematologic and solid organ malignancies as well as chronic systemic steroid use. Nevertheless, there are a limited number of reports of nocardial infection of immunocompetent individuals described in the literature [11–20]. Structural lung abnormalities such as bronchiectasis and COPD have been shown to be associated with nocardial infection among immunocompetent individuals [11–14].

The present report describes the clinical presentation of* N. beijingensis* as an endobronchial mass in an immunocompetent middle aged woman, without evidence of lung disease.

## 2. Case Presentation

A 55-year-old female presented to our hospital with a low grade fever, productive cough, and hemoptysis, which had developed over the previous 6 months.

Her past medical history included breast cancer, which was operated on without complications 9 years prior to her current admission. Based on symptoms, physical examinations, and imaging studies, she was diagnosed with community-acquired pneumonia and treated with several courses of doxycillin, amoxicillin, and cefuroxime. However, symptoms of low grade fever and cough persisted despite therapy.

The patient represented with an exacerbation of the fever and cough, now accompanied by progressive weight loss, and severe malaise having lost 5 Kg during the previous 6-month period.

On physical examination her temperature was 38°C, heart rate was 82 beats per minute, and her blood pressure was 142/84 mmHg. The patient's chest was clear to auscultation and no lymphadenopathy was present.

Laboratory studies demonstrated a hemoglobin (HB) level of 11.9 gr/dL, Hematocrit (HCT) of 36.6%, White Blood Cell (WBC) count of 21,820 K/*μ*L with 91.2% neutrophils, Erythrocyte Sedimentation Rate (ESR) of 85 mm/h, and C-Reactive Protein (CRP) of 14.2 mg/dL.

Serum liver enzymes and function tests were all within the normal limits. Evaluation of renal function revealed blood urea nitrogen (BUN) of 28 mg/dL, creatinine of 0.71 mg/dL, and normal urinalysis.

Serologic investigation for autoimmune disease revealed normal findings including anti-proteinase, anti-myeloperoxidase, anti-JO-1, anti-SCL, anti-SSA, anti-SSB, and anti-Smith antibodies, anti-nuclear antibodies, anti-double stranded DNA, and alpha-1 antitrypsin.

**Figure 1:**
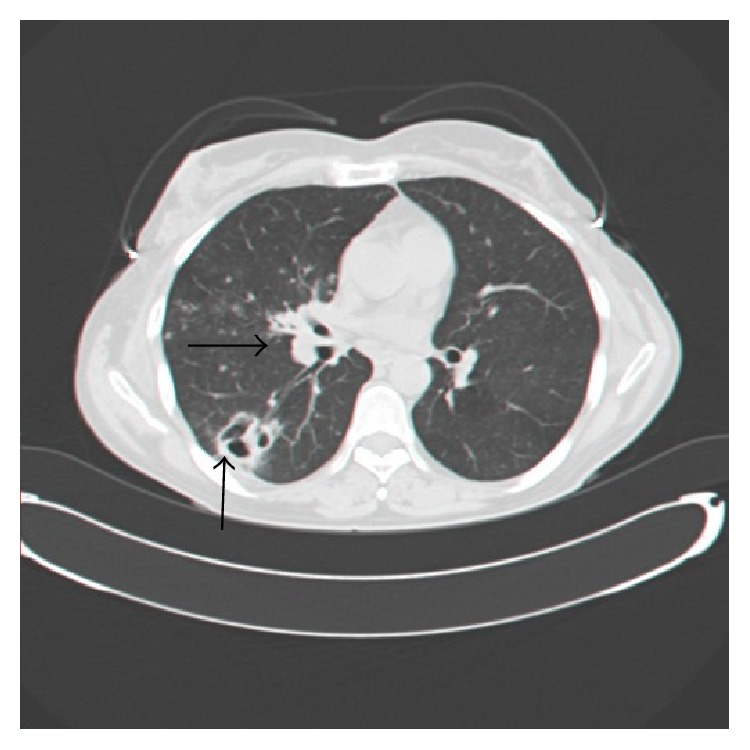
CT scan of the lung, axial view: the horizontal arrow is pointing toward nocardial mass in the right lower lob, while the longitudinal arrow is pointing toward nocardial cavitary lesion in the same lobe.

A computed tomography (CT) of her chest with contrast revealed an enlarged paratracheal lymph node of 15 mm, a space-occupying lesion with a diameter of 4.2 cm that externally compressed the right upper lobe, and cavitary lesion with a diameter of 3.2 cm in the right lower lobe (Figure 1).

Three bronchoscopies were performed over period of 3 months. The first bronchoscopy was performed on the 25th hospital day, a repeat bronchoscopy was performed 22 days later, and a third bronchoscopy was performed 30 days later.

The first bronchoscopy revealed white friable skipped lesions on the end bronchial surface of the right lower lobe. Multiple endobronchial and transbronchial biopsies were extracted and analyzed. A bronchoalveolar lavage (BAL) was also performed and fungal and bacterial cultures were obtained and plated on blood agar, chocolate agar, MacConkey agar, buffered charcoal yeast extract (BCYE) agar, and Lowenstein medium. Specimens were sent for Ziehl-Neelsen stain. The biopsy results showed granulation tissues with abscesses, mixed inflammation, and no signs of malignancy or granulomas. The bronchoscopic cultures were negative for pathogens.

Steroid therapy for a presumed vasculitic lesion was begun with prednisone 30 mg daily with a tapering dose until discontinued after 21 days. This yielded a brief general improvement at the first few days.

The patient returned after worsening of her symptoms and underwent a second bronchoscopy which did not present any additional information. She was discharged with antimicrobial empirical treatment with ciprofloxacin.

**Figure 2:**
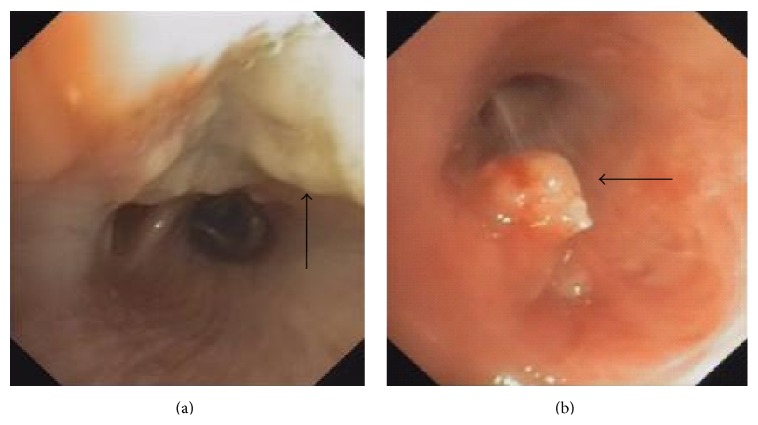
Bronchoscopic images: the arrows are pointing to different views of nocardial white friable lesions (a) and mass (b) in the right lower lobe.

In the following week, she was admitted again with high grade fever, coughing, weight loss, and general deterioration. A third bronchoscopy was performed which showed white friable material which was previously described (Figures 2(a) and 2(b)). We obtained viral (cytomegalovirus, adenovirus), bacterial (Legionella), and fungal cultures from the bronchoalveolar lavage. We also send the material for staining (PAS and silver stain) and performing PCR studies for* Pneumocystis carinii*,* Cryptococcus*,* Aspergillus*, and* Nocardia*. All of these lab examination revealed negative results. The only positive results were the encoded genes “RNA 16S” and “HSP 65” of the Actinomycetes family, consistent with* Nocardia beijingensis* which showed a 98% match.

**Figure 3:**
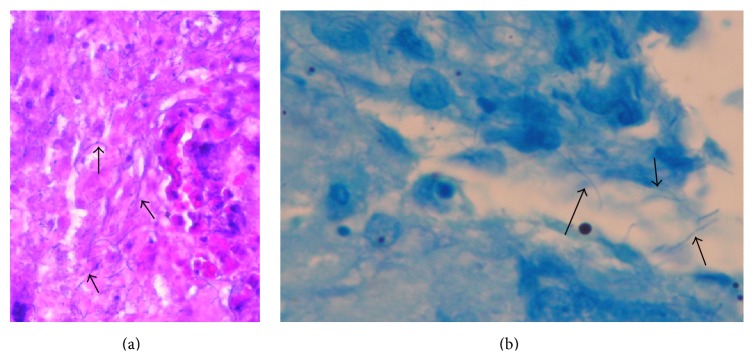
(a) Gram stain (×40). Arrows point toward Gram-positive filamentous microorganism. (b) Ziehl-Neelsen stain (×100). Arrows point toward partially acid-fast beaded branching filaments.

After several days of prolonged incubation,* Nocardia* colonies were visible. Antibiotic sensitivities showed the* Nocardia* species to be sensitive to all antibiotics and resistant only to ciprofloxacin (Table 1). A review of the bronchoscopic biopsy revealed Gram-positive filamentous microorganism, also confirming the diagnosis of* Nocardia* (Figure 3).

Thus, a diagnosis of endobronchial pulmonary nocardiosis was obtained and the patient was treated for 3 months with oral trimethoprim-sulfamethoxazole (TMP-SMX) and intravenous ceftriaxone for 1 month. Antibiotic treatment was followed by complete patient recovery.

## 3. Discussion

Nocardiosis is thought to be a rare, opportunistic disease. One would expect an increase in its prevalence due to immunosuppression and the increasing use of corticosteroids. In this report, we are able to demonstrate that the increased sensitivity of modern laboratory techniques enhanced our ability to detect nocardial infection even in a healthy individual.

Pulmonary nocardiosis in immunocompetent patients is the subject of a number of recent reports [11–20]. Although some of these patients had underlying lung abnormalities such as COPD or asthma, they did not receive therapy with immunosuppressives or steroids [11–14]. These sporadic reports indicate chronic air flow obstruction to be a risk factor for pulmonary nocardiosis. What makes the present description unusual is that our patient neither received immunosuppressive therapy nor had any underlying lung disease [15–20].

The diagnosis of pulmonary nocardiosis is difficult to document. Precious time may elapse, and during that time the condition of the patient might deteriorate. The median time for diagnosis of pulmonary nocardiosis was 32–42 days, which may increase to 55 days with dissemination to the nervous system [28]. Diagnosis in our patient required a total of 51 days. Mortality due to pulmonary nocardiosis continues to be high, between 14 and 40%, and increases significantly when there is dissemination to nervous system [28–30].

Several factors contribute to difficulties of diagnosis. Firstly, fungal cultures are time-consuming process; typical colonies are usually seen after 3 to 5 days and may even take up to 4 weeks [31]. Thus, it is critical to notify the laboratory when nocardial infection is suspected so that appropriate measures may be taken to optimize recognition and recovery of the organism. Secondly, it has been reported that in up to half of pulmonary nocardiosis cases the diagnosis cannot be achieved by sputum alone, thereby requiring further assessment of bronchoalveolar lavage or other respiratory samples [32]. Thirdly, prescribing empirical antibiotics therapy can contribute to difficulties in isolating the organism, which can cause complications when further invasive assessments are needed. Fourthly, serology is usually not useful, as no single serological technique can detect all of the clinically relevant species. Moreover, the antibody response is usually impaired in immunocompromised patients [33]. Fifthly, diagnosis is extremely difficult since nocardiosis is a rare disease, not well known by clinicians in daily practice. Finally, the clinical and radiographic findings in pulmonary and disseminated nocardiosis are nonspecific and may be mistaken for a variety of other bacterial infections, including actinomycosis and tuberculosis, as well as fungal infections, malignancies, and other diseases.

Uttamchandani et al., reporting a series of 30 cases of pulmonary nocardiosis, demonstrated infiltrates in 23 patients located in the upper lobe mimicking tuberculosis [10]. In others reports, empirical treatment for pulmonary tuberculosis was actually begun [11, 27, 34].

*Nocardia beijingensis* was first isolated by Wang et al. from soil in a sewage ditch in China in 2001 [3]. In 2004, the first human infections were reported in Thailand and Japan [4]. In 2008, a case of cutaneous* N. beijingensis* in an immunocompetent host was reported in France [5]. In 2011, the first pulmonary case outside Asia was reported [7]. In 2014, the first pulmonary case in the Western Hemisphere was reported [8].

No prospective randomized trials have determined the most effective therapy for nocardiosis. In addition, it is unlikely that such trials will ever be performed due to the uncommon nature and diverse clinical presentation. Thus, the choice of antimicrobials is based upon retrospective experience, animal model investigation, and* in vitro* antimicrobial activity profiles [35].

Treatment regimens effective against* Nocardia* spp. include trimethoprim-sulfamethoxazole (TMP-SMX), amikacin, imipenem, and third generation cephalosporins (ceftriaxone and cefotaxime). However, antibiotic susceptibilities vary among isolates [36].

In our patient, the pulmonary lesion was rare presentation of an endobronchial nocardial mass. This presentation mimics similar mass lesions seen in granulomatous or neoplastic diseases.* Nocardia* has been described as an endobronchial mass in 7 previous reports [21–27] presented in Table 2. In 2 previous reports, masses occluded one of the lung lobes and cause even severe atelectasis [21, 22].

In conclusion, pulmonary nocardiosis should be considered in the differential diagnosis of unresolving pneumonia or an endobronchial mass lesion in an immunocompetent individual. The diagnosis of an endobronchial mass lesion due to nocardial infection is rare and may be easily confused for tuberculosis or bronchogenic tumor. Appropriate tests need to be expeditiously obtained to document the diagnosis and beginning of therapy with an appropriately sensitive antibiotic such as trimethoprim-sulfamethoxazole. Prompt initiation of therapy is required to prevent central nervous system dissemination and increased patient morbidity and mortality.

## Figures and Tables

**Table 1:** *N. beijingensis* isolate antimicrobial susceptibility results.

Antibiotic	Susceptibility
Amikacin	Sensitive
Ciprofloxacin	Sensitive
Ceftriaxone	Sensitive
Imipenem	Sensitive
Minocycline	Sensitive
Sulfamethoxazole/trimethoprim	Sensitive
Ertapenem	Sensitive

**Table 2:** Summary of pulmonary nocardiosis cases presented as endobronchial mass.

Number	Age/sex	Smoking status	Clinical presentation	CXR/CT	Bronchoscopic findings	Identified species	Main treatment
1	73/male	Ex-smoker	Cough, fever, malaise, night sweats, and weight loss	Air space opacity RUL	Polypoid mass at the RUL [21]	*Nocardia asteroides*	TMP-SMX therapy, for 6 months

2	51/male	Ex-smoker	Malaise, low grade fever, chills, and cough	Infiltrate in the anterior segment of RUL	White exophytic lesion occluding the anterior segment RUL [22]	*Nocardia asteroides*	TMP-SMX therapy, for 3 months

3	28/male	Nonsmoker	Cough, fever, malaise, weight loss, night sweats, and dyspnea	Paramediastinal mass occluding RMB	Large fungating mass extending from the RMB [23]	*Nocardia asteroides*	Triple-sulfa therapy, for 6 months, gentamicin, for 3 months. RUL lobectomy

4	56/male	Ex-smoker	Cough, night sweats, and malaise	Left lung infiltrate	Mucosal edema and endobronchial mass [24]	*Nocardia asteroides*	Sulfisoxazole therapy, for 1 year

5	32/female	Unspecified	Fever, cough, and hemoptysis	RUL thick wall cavity with suspected fungal ball inside [25]	No bronchoscopy, on thoracotomy, fungal ball on RLL segments	*Nocardia* sp.(unspecified)	RML and RLL resection(unspecified antibiotics)

6	70/male	Smoker	Cough, dyspnea, anorexia, and weight loss	Mass in the RUL bronchus	Obstructing “tumor” of the RMB [26]	*Nocardia asteroides*	Minocycline, for 10 months

7	25/female	Nonsmoker	Persistent cough, pleuritic chest pain, and hemoptysis	Infiltrates RUL, RML, and RLL pleural effusion	Friable lesion “pearly white” occluding the entire segment [27]	*Nocardia* sp.(unspecified)	Antituberculosis medication. TMP-SMX therapy (unspecified duration)

8	55/female	Ex-smoker	Cough, weight loss, and hemoptysis	Endobronchial mass and cavitary lesion	Friable weight material, o*ur case*	*Nocardia beijingensis*	TMP-SMX therapy, for 3 months, ceftriaxone, for 1 month

RUL, right upper lobe; RML, right middle lobe; RLL, right lower lobe; RMB, right middle bronchus of lung; TMP-SMX, trimethoprim-sulfamethoxazole.

All patients had symptoms resolution after initiating the appropriate treatment, except in case 5 where the patient died due to late diagnosis.
