# Erratum to “Endobronchial Enigma: A Clinically Rare Presentation of *Nocardia beijingensis* in an Immunocompetent Patient”

**DOI:** 10.1155/2016/1950463

**Published:** 2016-07-17

**Authors:** Nader Abdel-Rahman, Shimon Izhakian, Walter G. Wasser, Oren Fruchter, Mordechai R. Kramer

**Affiliations:** ^1^The Pulmonary Institute, Rabin Medical Center, Beilinson Hospital, 49100 Petah Tikva, Israel; ^2^The Sackler Faculty of Medicine, Tel Aviv University, 69978 Tel Aviv, Israel; ^3^Mayanei HaYeshua Medical Center, 51544 Bnei Brak, Israel; ^4^Rambam Health Care Campus, 3109601 Haifa, Israel

In the article titled “Endobronchial Enigma: A Clinically Rare Presentation of* Nocardia beijingensis* in an Immunocompetent Patient,” [1] the name of the second author was given incorrectly as Shimon Izhakain. The author's name should have been written Shimon Izhakian. The revised author list is shown above.
